# Vanadyl Sulfate Treatment Stimulates Proliferation and Regeneration of Beta Cells in Pancreatic Islets

**DOI:** 10.1155/2014/540242

**Published:** 2014-08-19

**Authors:** Samira Missaoui, Khémais Ben Rhouma, Mohamed-Tahar Yacoubi, Mohsen Sakly, Olfa Tebourbi

**Affiliations:** ^1^Laboratory of Integrated Physiology, Faculty of Sciences of Bizerte, University of Carthage, 7021 Jarzouna, Tunisia; ^2^Department of Pathological Anatomy, Farhat Hached University Hospital, 4000 Sousse, Tunisia

## Abstract

We examined the effects of vanadium sulfate (VOSO_4_) treatment at 5 and 10 mg/kg for 30 days on endocrine pancreas activity and histology in nondiabetic and STZ-induced diabetic rats. In diabetic group, blood glucose levels significantly increased while insulinemia level markedly decreased. At the end of treatment, VOSO_4_ at a dose of 10 mg/Kg normalized blood glucose level in diabetic group, restored insulinemia, and significantly improved insulin sensitivity. VOSO_4_ also increased in a dose-dependent manner the number of insulin immunopositive beta cells in pancreatic islets of nondiabetic rats. Furthermore, in the STZ-diabetic group, the decrease in the number of insulin immunopositive beta cells was corrected to reach the control level mainly with the higher dose of vanadium. Therefore, VOSO_4_ treatment normalized plasma glucose and insulin levels and improved insulin sensitivity in STZ-experimental diabetes and induced beta cells proliferation and/or regeneration in normal or diabetic rats.

## 1. Introduction

Vanadium is a transition metal. It is estimated that more than 60 thousand tons of this element is emitted into the atmosphere each year as the result of human activities mostly from the combustion of fossil fuels [1].

After entering the circulatory system via gastrointestinal or respiratory tract, vanadium compounds are transported by transferrin or, less commonly, by albumin or low molecular components of plasma, such as citrates and, to a lesser extent, lactates or phosphates [2]. Many studies were conducted on inorganic and organic vanadium derivatives in induced diabetes animal models, in which the studied compounds were found to impact the levels of glucose, cholesterol, and triglycerides, with no significant harmful side effects upon prolonged administration [3–7]. Many experiments were also performed in diabetic patients, confirming the therapeutic effect of vanadium compounds on blood glucose levels with little toxic effects [8].

Vanadium (including vanadyl and vanadate) has been shown to reduce blood glucose level by stimulating glycogenesis, glucose uptake, and metabolism and by inhibiting glucose formation via hepatic gluconeogenesis and glycogenolysis [9, 10]. It has been found that vanadium and vanadium compounds exhibit an insulin-like activity [9, 11–13] by imitating insulin actions via insulin-receptor tyrosine kinase activation and kinase phosphorylation cascade pathways [14–17]. Therefore, vanadyl sulfate has been suggested as a therapeutic agent for the treatment of type 1 diabetes [12, 18–20]. Streptozotocin (STZ) treatment destroys the beta insulin-producing cells of the pancreas and STZ-induced diabetic rats are considered as a model of type 1 diabetes mellitus [21, 22]. Although vanadium compounds have been shown to have antidiabetic properties in STZ-induced diabetic model, the mechanism of their actions remained currently under investigation. The aim of this study was to investigate the responses of 30 days of treatment with vanadium sulfate in nondiabetic and STZ-induced diabetic rats.

## 2. Materials and Methods

### 2.1. Preparation of Diabetic Rats

The animals were made diabetic by an intraperitoneal injection (ip) of STZ in a single dose of 65 mg/Kg in 0.01 M citrate buffer (pH 4.5). This ip method was chosen based on recent reports which demonstrated a pronounced glucose increasing effect in STZ-diabetic rats [23, 24].

### 2.2. Animals and Treatment

Male Wistar rats, 5-6-week-old (weighting 175–200 g), were purchased from Pasteur Institute of Tunisia and used in accordance with the Local Ethic Committee of Tunis University for the use and care of animals in accordance with NIH recommendations. They were provided with food (standard pellet diet-Badr Utique-TN) and water ad libitum and housed five per cage under collected temperature (22°C) with a 12-hour light-dark cycle. The rats were divided into six groups.

Group 1 was nondiabetic control animals (ND control) and received daily an intraperitoneal injection (ip) of NaCl 9‰. Groups 2 and 3 were ND and received daily a dose of either 5 or 10 mg of VOSO_4_/Kg, respectively (ND + 5 mg/Kg and ND + 10 mg/Kg), for 30 days. Group 4 was a diabetic control (D control) injected with a single dose of 65 mg/Kg of STZ. Groups 5 and 6 comprised STZ diabetic animals treated with either 5 or 10 mg/Kg of VOSO_4_  during 30 days, respectively (D + 5 mg/Kg, D + 10 mg/Kg). STZ-groups were treated with VOSO_4_ after 48 hours of STZ-induced diabetes.

### 2.3. Biochemical Assays

All animals were fasted 12 hours before determination of glycemia by a glucometer (ACCU-CHEK-Active Roche). Insulin sensitivity was measured after ip injection of 1 U/Kg of insulin and blood glucose levels were determined at 30 min intervals during 2 hours as the percent of the hormone level at *t*_0_.

Animals were sacrificed after 24 hours of the last treatment and the blood was collected and serum was processed for estimation of insulin which was determined by Elisa kit (distributed by BioVendor).

### 2.4. Immunohistochemical Evaluation

Immunohistochemical staining of insulin was performed in order to characterize the integrity of beta cells in Langerhans islets. For this, tissue sections showing the maximum larger surface of Langerhans islets were selected for comparisons between groups. Sections were firstly deparaffined, hydrated through a decreasing gradual ethanol series, immersed in tampon citrate (pH 6) for 40 min, and then incubated with a polyclonal Guinea Pig anti-insulin antibody diluted in 50 mM Tris-HCl, pH 7,6 (DAKO). After washing, sections were incubated with phosphatase conjugated anti-Guinea Pig antibody and the procedure of revelation was assisted by TechMate 500 machine. Finally, sections were stained with haematoxylin/eosin.

Ten pancreatic islets per rat were examined under the microscope and the number of insulin-positive and insulin-negative cells was counted by numbering their nuclei. Data are expressed as the percent of insulin immunopositive cells/total number of cells in each islet.

### 2.5. Statistical Analysis

Significance of difference among various groups was evaluated by one-way analysis of variance (ANOVA) followed by Tukey's Multiple Comparison test, and *P* value was taken as significant at 5% level.

## 3. Results

**Figure 1:**
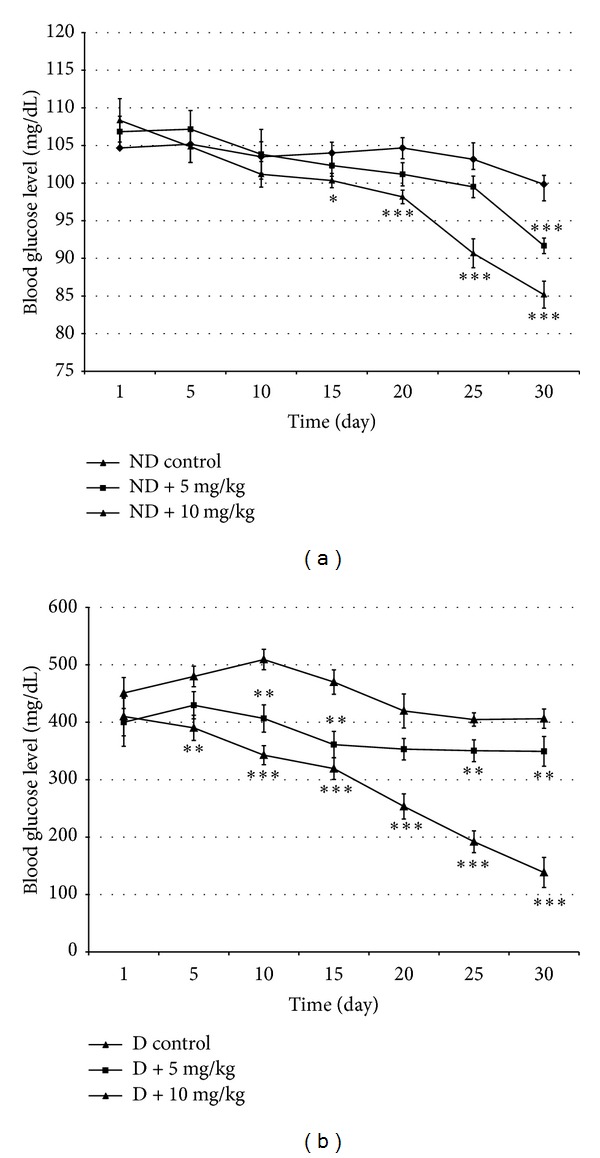
Effects of vanadyl sulfate on blood glucose level in normal (a) and STZ-diabetic (b) rats. Values are mean ± SD. *P < 0.05; ***P < 0.001 versus corresponding control. *N* = 10 per group.

The effect of VOSO_4_  treatment on the blood glucose levels in nondiabetic (ND) and diabetic (D) rats is shown in Figure 1. There were no significant changes in the blood glucose levels in control ND group during the treatment (Figure 1(a)). Treatment of ND rats with VOSO_4_  at 5 or 10 mg/Kg did not change significantly the blood glucose levels during the first 10 days. However, blood glucose level decreased significantly at the end of treatment with the dose of 5 mg/Kg and was significantly reduced from 15 to 30 days with the dose of 10 mg/Kg as compared to control ND group. At day 30, the glycemia decrease was about 8 and 15%, respectively, for the doses of 5 and 10 mg/Kg compared to control group. As suspected, a notable increase of blood glucose level was observed in the STZ-treated control rats compared to untreated animals (406.25 ± 16.70 versus 99.83 ± 5.19 mg/dL) indicating diabetes installation that was maintained during all experiment. Treatment of diabetic group (D) with 5 mg/Kg of VOSO_4_  reduced significantly blood glucose level from day 10 while a dose of 10 mg/Kg decreased blood glucose level significantly and gradually from day 5 to reach normal level at day 30. The noted values of glycemia at 30 days were 349.40 ± 16.83 and 138.50 ± 6.19 versus 406.25 ± 16.70 mg/dL, respectively, in D + 5, D + 10, and control D groups (Figure 1(b)).

Table 1 showed the levels of insulin in ND and D groups without and following VOSO_4_  treatment. The ND rats treated with 5 mg/Kg of VOSO_4_ did not show significant changes in insulinemia level, but a marked increase was obtained with 10 mg/Kg compared to control ND group (2.76 ± 0.07 versus 1.73 ± 0.04 *µ*g/L). Moreover, in control D group, insulinemia was profoundly decreased in comparison to control ND animals (0.07 ± 0.01 versus 1.73 ± 0.04 *µ*g/L). Administration of VOSO_4_ in D groups markedly increased the insulinemia in a dose-dependent manner. In fact, insulin levels were increased in diabetic rats from 0.07 ± 0.01 to 0.49 ± 0.004 and 1.26 ± 0.12 *µ*g/L with, respectively, 5 mg and 10 mg/Kg doses.

**Figure 2:**
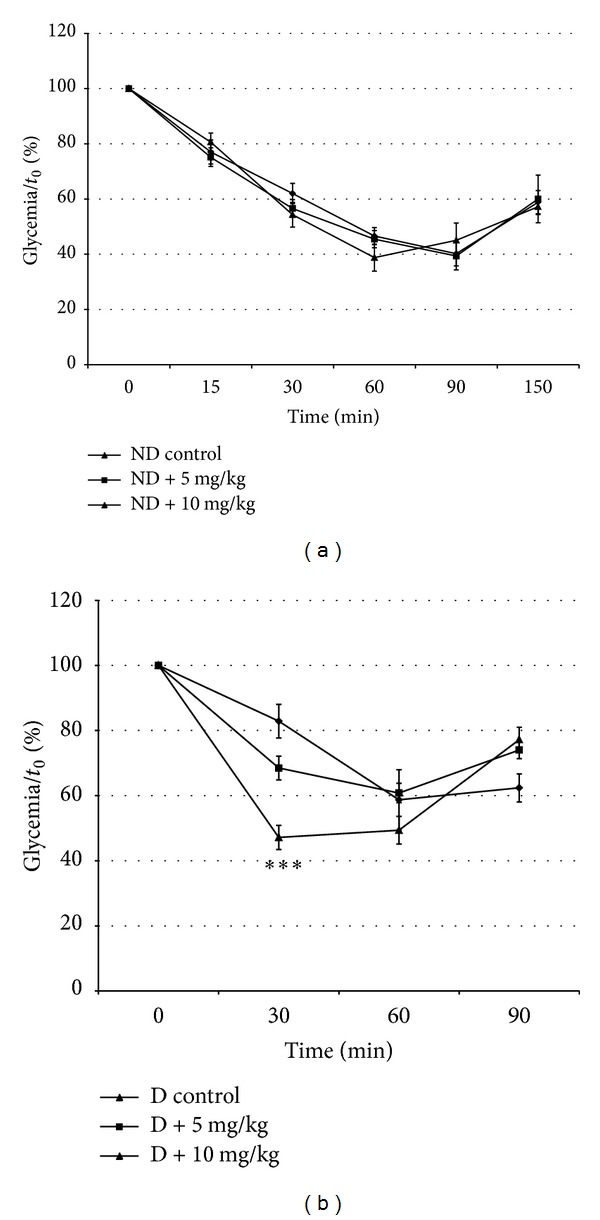
Effect of vanadyl sulfate on the insulin sensitivity in normal (a) and STZ-diabetic (b) rats. Values represent the percentage change from glycemia at *t*_0_. Values are ±SD. **P < 0.01; ***P < 0.001 versus corresponding control. *N* = 10 per group.

Insulin sensitivities changes following the ip injection of insulin (1 U/Kg) in normal and diabetic treated animals are shown in Figure 2. In ND rats, treatment with 10 or 5 mg of VOSO_4_  did not alter the glycemia profile in response to insulin administration (Figure 2(a)). Indeed, in these groups, the maximum hypoglycemic effect of insulin was observed by 60 and 90 min, respectively, and was about 40% of initial glycemia value. Diabetic animals treated with the low dose of VOSO_4_ showed a similar glycemia profile than control D group with a maximum decrease by 60 min while those treated with the high dose exhibited a significant (50%) and rapid maximum decrease of glycemia by 30 min after insulin injection (Figure 2(b)).

**Figure 3:**
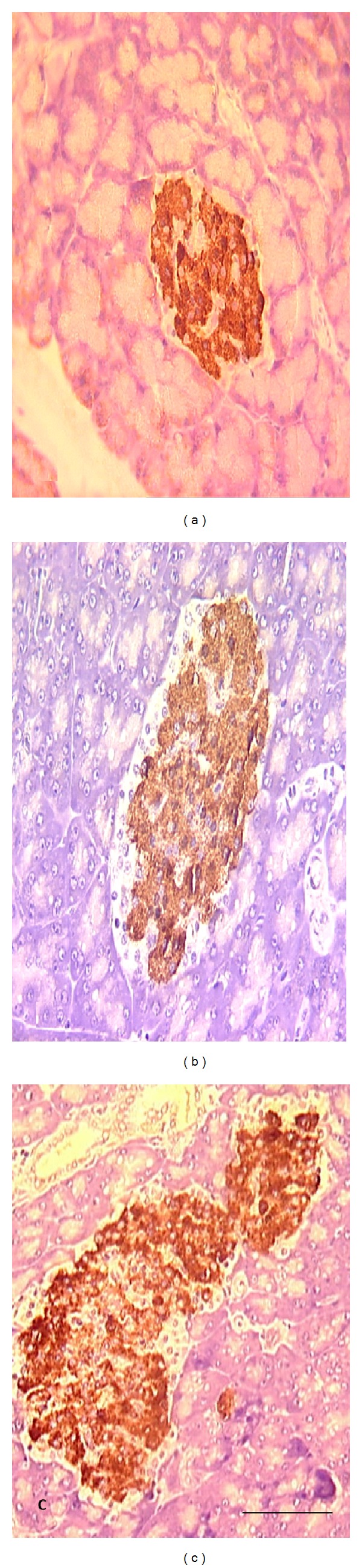
Immunohistochemical staining of insulin in pancreatic Langerhans islet beta cells from nondiabetic rats. Note that insulin immunopositive cells are greater in (b) and more in (c). Control ND (a), ND treated with 5 mg/Kg of VOSO_4_ (b), and ND treated with 10 mg/Kg of VOSO_4_ (c). Six rats were used per group and 10 islets per rat were analyzed. Scale bar: 50 *µ*m.

**Figure 4:**
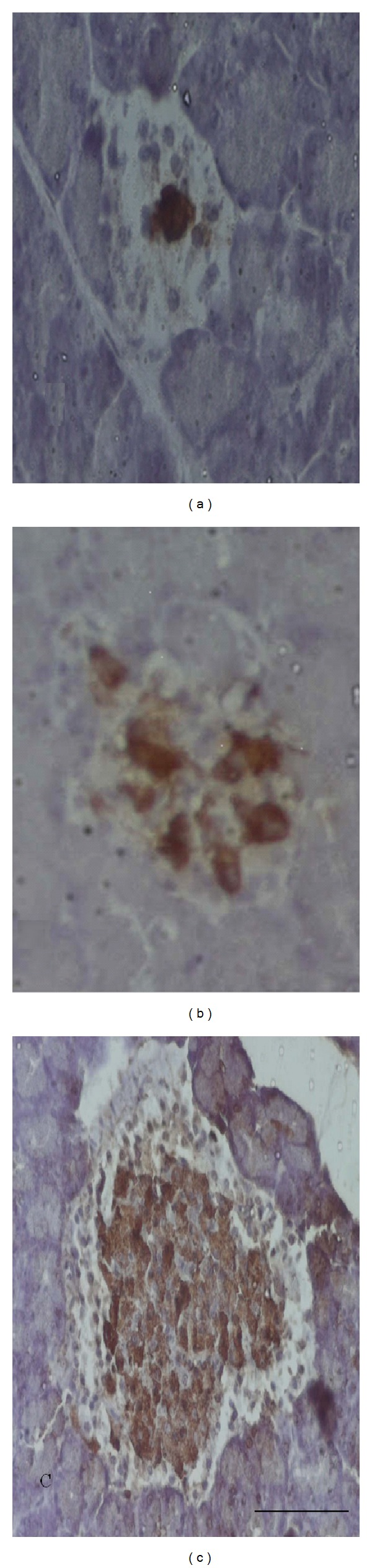
Immunohistochemical staining of insulin in pancreatic Langerhans islet beta cells from STZ-diabetic rats. Note that there are more insulin immunopositive cells in the treated STZ-diabetic rats than in nontreated STZ-diabetic rats. Control D (a), D treated with 5 mg/Kg of VOSO_4_ (b), and D treated with 10 mg/Kg of VOSO_4_ (c). Ten islets per rat were analyzed. Scale bar: 50 *µ*m.

**Figure 5:**
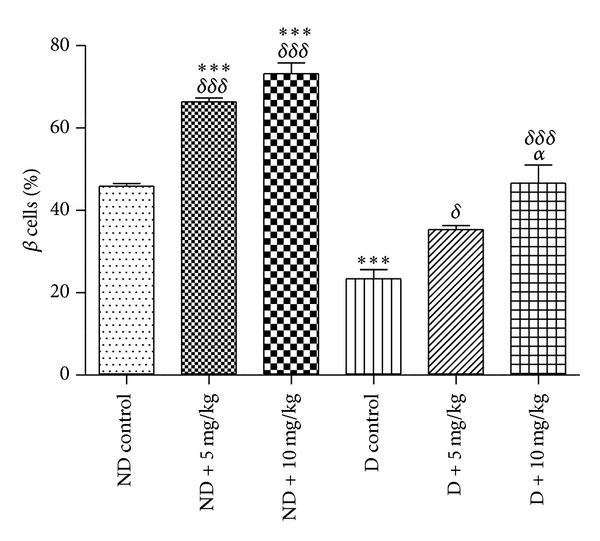
Effect of vanadyl sulfate on the number of beta cells in %. Values represent mean ± SD. *P < 0.05; ***P < 0.001 versus ND control. ^*δ*^ *P* < 0.05; ^*δδδ*^ *P* < 0.001 versus D control. ^*α*^ *P* < 0.05 versus D + 5 mg/Kg. 10 islets per rat were analyzed.

In VOSO_4_  treatment groups, the size of pancreatic islets was observed to be larger compared to their respective control ND and D groups (Figures 3 and 4). Statistical analyses showed that VOSO_4_  treatments induced a marked increase in the number of insulin immunopositive cells by about 20% in ND + 5 and 27% in ND + 10 mg/Kg groups (Figure 5). VOSO_4_  treatment increased significantly the number of insulin immunopositive cells in the STZ-diabetic groups in a dose-dependent manner by 12 and 23%, respectively, with 5 and 10 mg/Kg doses (Figure 5). Comparison between STZ-diabetic treated groups and ND control rats indicated no significant differences.

## 4. Discussion 

We studied the antidiabetic properties of vanadium by exploring its effects on blood glucose and insulin levels as well as B cells of endocrine pancreas.

Using STZ-induced diabetic rats, we showed that treatment with vanadyl sulfate (VOSO_4_) at 5 and 10 mg/Kg significantly reduced the mean blood glucose levels by the 10th and 5th days, respectively, in comparison with untreated control group. At day 30, diabetic rats given the higher dose of the compound exhibited similar glycemia level than ND control animals. However, in ND rats, treatment with the lower dose of VOSO_4_ did not change significantly the blood glucose level while the higher dose induced little and gradually decrease by the day 15 of treatment and the glycemia was decreased by about 14% at day 30. Our results are in agreement with previous data demonstrating that oral administration of vanadium compounds in STZ-induced or genetically inherited or nutritional diabetic animals significantly ameliorated hyperglycemia [25–29]. In humans, Soveid et al. [30] reported the safety and the efficacy of oral vanadyl sulfate therapy during 30-month period in type 1 diabetic patients by decreasing insulin need and blood glucose level.

Our study also revealed that the hypoglycemic effect of VOSO_4_  was accompanied by a marked increase of basal insulinemia level in diabetic group in a dose-dependent manner. With the dose of 10 mg, the insulinemia level of diabetic rats was almost normalized. Indeed, the decrease of blood glucose levels registered particularly in diabetic rats exposed to vanadium compound may be related to the concomitant increase of plasma insulin concentrations. These results were in accordance with previous data concerning the effectiveness of vanadium compounds in ameliorating diabetic state [30–32].

Further, we have investigated the effect of vanadium administration on insulin sensitivity in diabetic and nondiabetic rats. In ND groups, we showed a maximum hypoglycemic effect of insulin at 60–90 min after hormone introduction. However, in D groups, VOSO_4_  treatment with a dose of 10 mg enhanced insulin sensitivity with a more rapid and pronounced decrease of blood glucose following hormone injection while the dose of 5 mg did not change significantly the glycemia response of D rats. The improved insulin sensitivity induced by VOSO_4_ is in line with numerous studies indicating that vanadium can be considered as a potent insulin mimetic or insulin trophic in various tissues [24, 33].

It has been shown that vanadium compounds are characterized by multiple ways of action resulting in blood sugar decrease [34].* In vivo* and* in vitro* studies reported that vanadyl sulfate increased glucose transport and metabolism in skeletal muscles, liver, and adipose tissues [25]. Thanks to their structural similarity to orthophosphate anions, the vanadium organic derivatives inhibit protein phosphotyrosine phosphatase [35]. They also inhibit the activity of PTP-1B, enzyme responsible for the dephosphorylation of insulin receptor, causing insulin resistance [36, 37]. Another mechanism of reduction of blood glucose levels by vanadium compounds is the activation of PKB/Akt kinase leading to the increase of glucose uptake by the GLUT4 transporter [34, 38]. Activation of PKB/Akt stimulates also the phosphorylation of GSK3, resulting in the stimulation of glycogen synthesis [34, 39]. On the other hand, it was found that inhibition of C-jun N-terminal kinase improves insulin sensitivity in experimental diabetes [40].

Vanadium increases glucose transport and oxidation and insulin-receptor tyrosine-kinase activity and exerts insulin-like effects on glucose and lipid metabolisms by insulin-dependent or insulin-independent biochemical pathways [9, 10, 41].

It is well known that STZ treatment destroys beta cells of pancreatic islets inducing insulin-dependent diabetes mellitus [19, 22]. Thus, in the present study, we showed a reduction in the area of pancreatic islets associated with a marked decrease in the number of immunoreactive beta cells for insulin in diabetic group in comparison with nondiabetic control animals. However, in both ND and D groups, VOSO_4_ treatment enlarged the size of pancreatic islets in comparison with respective control groups. Most importantly, VOSO_4_  treatment of diabetic rats increased in a dose-dependent manner the number of insulin immunopositive beta cells as distinguished from diabetic untreated group, suggesting that VOSO_4_ can generate the beta cells in STZ-induced diabetic rats. In fact, the number of beta cells in islets of diabetic VOSO_4_-treated rats with 5 and 10 mg was not significantly different from that of ND group. This is in agreement with previous investigations concerning the pancreatic insulinotropic property of this compound [18, 24, 42]. Interestingly, vanadium treatment also increased significantly beta cells number in islets of nondiabetic rats.

In a mouse model of pancreas alloxan-perfused segment, Waguri et al. [43] reported processes of beta cells regeneration from extra-islet precursor cells. Indeed, in STZ-diabetic rats, VOSO_4_ treatment might stimulate beta cells proliferation from intraislet endocrine cells and differentiation from extra-islet precursor cells.

In conclusion, the present study demonstrates that vanadyl sulfate could be useful as a potential antidiabetic agent. However, as the current evidences are still limited and based mostly on animal models and little is known about the therapeutic formulations and the side effects of vanadium compounds, further investigations on their long-term use as conventional therapy for diabetic patients are needed.

## Figures and Tables

**Table 1:** Effects of vanadyl sulfate on insulinemia in nondiabetic and diabetic rats. Values are mean of blood insulin levels ± SD (standard deviation).

Group	Nondiabetic			Diabetic		
VOSO_4_ mg/Kg	0	5	10	0	5	10
Insulinemia (*µ*g/L)	1.73 ± 0.04	1.97 ± 0.12^*δδδ*^	2.76 ± 0.07^∗∗∗*δδδ*^	0.07 ± 0.01∗∗∗	0.49 ± 0.04^∗∗∗*δ*^	1.26 ± 0.12^∗∗*δδδπππ*^
*n*	8	6	6	8	7	6

*n*: number of determination used in this experiment.

**P < 0.01; ***P < 0.001 versus ND control.

^*δ*^ *P* < 0.05; ^*δδδ*^ *P* < 0.001 versus D control.

^*πππ*^ *P* < 0.001 versus D + 5.
